# Special Issue “Advanced Materials for Gas Sensors”

**DOI:** 10.3390/ma14226765

**Published:** 2021-11-10

**Authors:** Cristian E. Simion

**Affiliations:** National Institute of Materials Physics, Atomistilor 405A, 077125 Magurele, Romania; simion@infim.ro

Today’s view on gas sensors end-users is more directed toward miniaturization, low power consumption, and intelligent device integration aiming to reply to several hot issues such as high sensitivity, optimum selectivity, fast response/recovery transients, and good long time stability. Spanning the literature related to the gas-sensitive materials, one has to admit that most of the work has been carried out via a trial-and-error approach.

Establishing sensor selective sensitivity for specific gases is challenging, and it depends on many parameters, such as gas adsorption, surface reaction kinetics, and charge transfer from the semiconductor. Thus, a fundamental understanding of the involved processes with specific gas-sensitive materials is of crucial importance for the future unveiling of the structure-functioning relationships.

To meet the increased demand for low power consumption gas-sensing devices, Lee et al. [1] prepared a hybrid composite gas sensor made of tin oxide and reduced graphene oxide with good sensing performances toward CO_2_ detection when operating at room temperature. The sensing mechanisms involved in the CO_2_ detection were described under a pure nitrogen atmosphere and in the presence of 50% relative humidity. It was demonstrated that based SnO_2_–rGO exhibit a sensor signal to 100 ppm CO_2_ of 6.7 times higher than that of simply reduced graphene oxide. The gas interaction of the SnO_2_–rGO hybrid composite was attributed to the high conductivity and active sites of p–n heterojunctions.

Another study [2] focused on H_2_S detection under the presence of humidity using CuWO_4_-based gas sensors. A comprehensive study was conducted in order to explain the major role played by the native Cu(OH)_2_ surface layers within the sensing mechanism. Herein, the authors merged insights about structural, morphological, and surface chemistry with phenomenological gas-sensing investigations (via simultaneous electrical resistance and contact potential difference), aiming to highlight the way in which surface hydroxylation plays a major role toward H_2_S sensing. Importantly, a theoretical approach was developed in order to link the as-obtained sensing behavior with the intrinsic properties (in terms of Debye length).

Bratu et al. [3] analyzed the gases released by Golden Delicious apples over a defined period. By using CO_2_ laser photoacoustic spectroscopy, the researchers determined low traces of ethylene, ethanol, and ammonia. This is a straightforward investigation method able to provide realistic information about gas species in the downstream gas. According to their results, as the concentration of ethanol increases, ethylene production decreases, and ammonia concentration in the downstream gas increases. Although these outcomes were not connected with an appropriate sensing mechanism, they can be further linked to postharvest fruit management.

In the paper [4], the authors employed CeO_2_:Mn_3_O_4_ mesoporous micro-pellistors toward CH_4_ detection under the presence of 50% relative humidity. It was found that besides porosity, also the molar ratio between CeO_2_ and Mn_3_O_4_, exhibits a major contribution toward CH_4_ combustion temperature. Based on the correlation with the additional investigations such as XPS, TPR, and Raman, we found that a few important factors merge toward enhancing the catalytic behavior. These are high surface-to-volume ratio, oxygen vacancies formation, and increased reducibility. It was demonstrated that CeO_2_:Mn_3_O_4_ with a 7:3 molar ratio exhibited a parabolic dependence of the catalytic behavior with respect to the CH_4_ concentration. A schematic approach of the involved phenomena was addressed, aiming to follow the logical pathway of the catalytic mechanism.

In the work of Ramanovičius et al. [5], non-stoichiometric WO_3_-based gas sensors were tested toward different volatile organic compounds such as methanol, ethanol, isopropanol, acetone, and additionally to ammonia. Knowing that the operating temperature plays an important role with respect to sensitivity, selectivity, and the associated kinetics, and further transduced in response/recovery transients, the authors tested WO_3_ sensors over a wide range of temperatures—spanning from 60 °C up to 270 °C. According to their results, apart from the fact that the analytical sensor signals vary with the operating temperature, an additional dependence feature was observed in relation to the structural properties. The main outcome of this work is reflected through the low operating temperature of WO_3_/WO_3−x_-based gas sensors with optimum sensitivity and selectivity performances when aiming to detect volatile organic compounds and ammonia. Seen in perspective, this may open new ways for mass production of gas sensors array using WO_3_-based materials.

An enhanced catalytic conversion process of acetone with Pd-loaded SnO_2_ was proved by the work of Gschwend et al. [6]. Knowing that Pd addition to SnO_2_ may enhance or downgrade the sensing performances of based SnO_2_ is of crucial importance to tune the desired amount in such a way that maximum sensitivity is attained. Accordingly, PdO_x_ clusters with diameters ranging from 4 to 6 nm were found on the SnO_2_ surface. In addition, the best operating temperature toward acetone detection decreases with increasing Pd loading. A reverse behavior favor sensing response and recovery times. Based on the spill-over effect, the sensitization effect was explained, and the activation energies were calculated based on the reaction rates.

Solid-state sensors and laser photoacoustic spectroscopy have proven the ability to detect a wide variety of target gases and can be used in many different applications.

The papers published in this Special Issue have already earned a few citations, thus starting to gain readers’ attention. It is foreseen that further investigations on the presented materials are strongly related to material preparation strategies to boost the overall knowledge of gas sensing phenomena. This is the driving force of initiating the Special Issue “Advanced Materials for Gas Sensors (Volume II)” with a high scientific impact on the interdisciplinary research.
